# Which model is superior in predicting ICU survival: artificial intelligence versus conventional approaches

**DOI:** 10.1186/s12911-022-01903-9

**Published:** 2022-06-26

**Authors:** Farzad Mirzakhani, Farahnaz Sadoughi, Mahboobeh Hatami, Alireza Amirabadizadeh

**Affiliations:** 1 Department of Health Information Management, School of Health Management and Information Sciences, Iran University of Medical Science, No. 4, Rashid Yasemi Street, Vali-e Asr Avenue, Tehran, 1996713883 Iran; 2 Antimicrobial Resistance Research Center, Communicable Disease Institute, Mazandaran University of Medical Sciences, Sari, Iran; 3 Endocrine Research Center, Shahid Beheshti University of Medical Sciences, Tehran, Iran

**Keywords:** Critical care, Prognostic models, Artificial intelligence, Machine learning, Decision tree, Artificial neural network, SOFA, SAPS, APACHE

## Abstract

**Background:**

A disease severity classification system is widely used to predict the survival of patients admitted to the intensive care unit with different diagnoses. In the present study, conventional severity classification systems were compared with artificial intelligence predictive models (Artificial Neural Network and Decision Tree) in terms of the prediction of the survival rate of the patients admitted to the intensive care unit.

**Methods:**

This retrospective cohort study was performed on the data of the patients admitted to the ICU of Ghaemshahr’s Razi Teaching Care Center from March 20th, 2017, to September 22nd, 2019. The required data for calculating conventional severity classification models (SOFA, SAPS II, APACHE II, and APACHE IV) were collected from the patients’ medical records. Subsequently, the score of each model was calculated. Artificial intelligence predictive models (Artificial Neural Network and Decision Tree) were developed in the next step. Lastly, the performance of each model in predicting the survival of the patients admitted to the intensive care unit was evaluated using the criteria of sensitivity, specificity, accuracy, F-measure, and area under the ROC curve. Also, each model was validated externally. The R program, version 4.1, was used to create the artificial intelligence models, and SPSS Statistics Software, version 21, was utilized to perform statistical analysis.

**Results:**

The area under the ROC curve of SOFA, SAPS II, APACHE II, APACHE IV, multilayer perceptron artificial neural network, and CART decision tree were 76.0, 77.1, 80.3, 78.5, 84.1, and 80.0, respectively.

**Conclusion:**

The results showed that although the APACHE II model had better results than other conventional models in predicting the survival rate of the patients admitted to the intensive care unit, the other conventional models provided acceptable results too. Moreover, the findings showed that the artificial neural network model had the best performance among all the studied models, indicating the discrimination power of this model in predicting patient survival compared to the other models.

## Background

The safest place for critically ill patients is the Intensive Care Unit (ICU). The ICU is a specialized unit in which advanced medical technologies are used by the most experienced staff. Improved patient survival, better quality services, and the patient’s well-being and convenience are the most important goals of the ICU [1]. The mortality rate of patients admitted to the ICUs can range from 7 to 52.3% and the prognosis is completely different [2, 3]. It is because critical care usually needed immediate high-risk decision-making in the situation of uncertainty, and the patient outcome is related to various factors, such as the admission type, age, chronic diseases, acute physiological changes, etc. [4]. Therefore, real-time decision making based on the numerous and various amount of data in the ICUs is one of the most important challenges faced by clinicians. It strongly depends on efficiency of clinicians. In addition, the lack of stability of ICU admitted patients’ illness and their frequent need for dangerous interventions and medications lead to the higher rates of patient safety events than other hospital wards [5, 6].

The prediction of patient survival in the ICU is useful in supporting clinical and managerial tasks such as appropriate treatment planning, optimal resource allocation, determining workload, and evaluating the quality of care, and it can play an important role in providing deeper insights into the health status of patients and the ICU management for the clinical staff and managers of this ward [7, 8].

In recent decades, several models have been developed for predicting patient survival in the ICU. These models mainly calculate the probability of patient survival by determining the severity of the disease based on the physiological symptoms of the patient [9–11]. Some of these prediction models are SOFA, SAPS II, APACHE II, and APACHE IV. These models are based upon physiological disorders and are used to measure the degree of physiological instability and the severity of abnormalities in the body’s vital organs. They seek to quantify functional disorders, which can promote a common understanding of the patient conditions and help the clinical staff develop quality control programs related to patient care [7].

The prediction model SOFA is used to describe the status of patients in the ICU. This tool assesses the status of the body organs systematically and continuously during the patient’s stay in the ICU. In this model, a score, from zero to four, is assigned to the functioning of each of the six organs (respiratory, cardiovascular, hepatic, coagulation, renal and nervous). A score of zero means no disorder and a score of four means severe organ dysfunction. Thus, the sum of numerical indices will be from zero to 24 [12]. The model SAPS II uses 17 variables (12 physiological variables, age, type of admission, and three variables related to underlying diseases) so as to calculate the probability of patient survival in the ICU. The total score obtained from each of these variables is a number between zero and 163, and the higher the score, the higher the risk of death [13]. APACHE II uses the 12 physiological variables along with age and chronic diseases of the ICU patient to calculate survival probability. The range of scores for this numerical model is between zero and 71, and the higher the score, the higher the probability of death [14]. APACHE IV model was developed through the reformulation of APACHE III equations. This model predicts patient survival by collecting minimum and maximum values for each variable in the first 48 h of ICU admission [15, 16]. Table 1 presents the variables for each model.

**Table 1:** List of variables collected in conventional severity classification systems (SOFA, SAPS II, APACHE II and APACHE IV)

Variable Name	SOFA	SAPS II	APACHE II	APACHE IV
Age		✔	✔	✔
Glasgow coma score (GCS)	✔	✔	✔	
Eyes response				✔
Verbal response				✔
Verbal response				✔
Body temperature (TEMP)		✔	✔	✔
Systolic blood pressure (SBP)		✔		
Diastolic blood pressure (DBP)				✔
Mean arterial pressure (MAP)	✔		✔	✔
Heart rate per minute (HR)		✔	✔	✔
Arterial pH (PH)			✔	✔
Level of carbon dioxide in the arterial blood (PCO2)				✔
FIO2	✔	✔	✔	✔
Respiratory rate per minute (RR)			✔	✔
Level of oxygen in the arterial blood (PAO2)	✔	✔	✔	✔
Urinary output	✔	✔		✔
Serum creatinine (Cr)	✔		✔	✔
Serum sodium (Na)		✔	✔	✔
Serum potassium (K)		✔	✔	
Blood urea nitrogen (BUN) or urea level		✔		✔
Bicarbonate (HCO3)		✔		
Bilirubin level (BIL)	✔	✔		✔
Albumin (ALB)				✔
Blood sugar level (BSL)				✔
White blood count (WBC)		✔	✔	✔
Platelets (PLT)	✔			
Mechanical ventilation use	✔	✔	✔	✔
Vasopressors	✔			
Hematocrit (Hct) (%)			✔	✔
Chronic diseases/morbidities		✔	✔	✔
Others (Type of admission, 30-day readmission, Pre-ICU LOS, etc.)		✔	✔	✔

All the above-mentioned models are among the conventional prediction models which have been developed using statistical methods. In modeling the relationships between variables, statistical methods have some assumptions and limitations. In addition, these methods lack the capability of modeling complex nonlinear relationships and high-degree interactions [17]. Therefore, there is a need to adopt less restrictive methods. In recent years, the use of new methods of data processing and analysis, such as Artificial Intelligence (AI), has received much attention in biomedical research. Classification and prediction are among the most important applications of AI methods in various sciences. AI can greatly help disease management as it can identify and diagnose diseases, categorize patients, and predict the prognosis and outcome of diseases. Among the most common and widely used methods of AI, one can mention Artificial Neural Network (ANN) and Decision Tree (DT) [18].

So far, many studies have evaluated SOFA, SAPS II, APACHE II, and APACHE IV in the ICU. Some studies have also evaluated the ANN and DT models separately so as to predict the survival of the patients admitted to the ICU. This being said, the present study compares conventional prediction models (SOFA, SAPS II, APACHE II, and APACHE IV) with modern ones (ANN and DT) and evaluates them in terms of the prediction of the survival of the patients admitted to the ICU.

### Related work

Zhang et al. developed a decision tree model for predicting the outcome of patients with community-acquired pneumonia in the ICU and compared it with two conventional models, CURB-65 and SOAR. In this study, which was performed on the MIMIC III database, 3519 patients participated. The area under the ROC curve (AUROC) for CART DT, CURB-5, and SOAR models were 0.661, 0.609, and 0.589, respectively, showing that the decision tree performed better than the other two models in predicting patient survival [19].

In a study conducted by Sharma et al., three models (SOFA, SAPS II, and APACHE II) were compared in terms of the prediction of mortality in patients with sepsis. The ROC analysis showed that the best discrimination power belonged to SAPS II (AUROC = 0.981), followed by APACHE II (AUROC = 0.978) and SOFA (AUROC = 0.911). The findings of this study show that SAPS II performs better than APACHE II and SOFA in predicting the survival of patients with sepsis, but it is necessary to take into account a combination of factors when estimating the survival prediction in the ICU [20].

In a study by Nimgaonkar et al., APACHE II and an artificial neural network were compared in terms of the prediction of patient survival in an ICU in India. The data of 1962 patients were used for training, and the rest of the data were used to test the artificial neural network. In this study, which used the area under the ROC curve criterion to evaluate the discrimination power of the models, the findings showed that the discrimination power of the artificial neural network was better than that of APACHE II (0.87 vs. 0.77). In addition, the results of model calibration showed that the calibration in the neural network was better than the calibration in APACHE II [21].

In their article entitled "a comparative study of the performance of SOFA and APACHE II scoring systems at the admission time in determining the prognosis of patients with trauma in the intensive care unit", Kashefi et al. carried out a retrospective, cross-sectional descriptive study on 100 patients. The results of the study showed that the sensitivity and specificity of APACHE II were 97.4 and 36.1%, and the sensitivity and specificity of SOFA were 97.4 and 16.4%, respectively. The study concluded that these two models were effective in predicting the survival of trauma patients admitted to the ICU [22].

## Methods

### Study design

This retrospective cohort study was performed on the data of the patients admitted to the ICU of Razi Educational and Medical Center of Ghaemshahr from March 2017 to September 2019. As for the inclusion and exclusion criteria, the patients had to be above 18 years of age, burn patients, patients with coronary artery disease, heart surgery patients, brain dead patients, and patients with a stay of shorter than 24 h were excluded from the study.

### Data collection

The list of the variables comprises age, gender, date and time of admission to the ICU, date and time of discharge from the ICU, patient’s status at the time of discharge from the ICU (hospital mortality), type of admission (medical problem, elective surgery or emergency surgery), ICU admission diagnosis, underlying diseases, the dose of vasopressors prescribed, the state of consciousness (GCS), body temperature (TEMP), heart rate per minute (HR), respiration rate per minute (RR), systolic blood pressure (SBP), diastolic blood pressure (DBP), the level of blood acidity (PH), the level of carbon dioxide in the arterial blood (PCO2), the level of oxygen in the arterial blood (PAO2), ventilator use, FiO2, the level of urine output, blood sugar (BS), blood urea nitrogen (BUN), creatinine (Cr), sodium (Na), potassium (K), platelets (PLT), bilirubin (BIL), albumin (ALB), hematocrit (Hct) and white blood count (WBC). It should be noted that the collected data were related to the first 24 h of patient admission to the ICU, and for medical test variables, the minimum and maximum values in the patient’s medical record were collected.

### Conventional models: SOFA, SAPS II, APACHE II, and APACHE IV

To calculate the scores of the conventional models of SOFA, SAPS II, and APACHE II, an online hybrid calculator (https://clincalc.com/IcuMortality/Default.aspx) was used, and to calculate the score of the conventional model APACHE IV, another online calculator (http://www.icureach.com/icu_scores/apacheIV.php) was utilized. These scores were calculated for each patient on the first day of their stay in the ICU. Then, to evaluate the performance of these models, ROC analysis was performed on them by considering the variable *“hospital mortality”* as the dependent variable.

### AI models: multilayer perceptron neural network and CART decision tree

Univariate logistic regression analysis was used to select the variables for the models, and then the variables that had a statistically significant relationship (at the level of 0.05) with the outcome of *“hospital mortality”* as the dependent variable were entered into the AI models as the selected variables. Then, by randomly assigning 70% of the patients to the training set and 30% of the patients to the test set and by tuning the hyperparameters related to the ANN and the DT using the grid search method, AI models (MLP NNs and CART DTs) were developed and among them, the best model of an ANN and the best model of a DT, in terms of performance, were selected. In addition, five-fold cross-validation was utilized during model training to minimize overfitting. Moreover, four AI models, namely, the MLP NN model with all input variables (MLP NN_all_), MLP NN model with selected input variables (MLP NN_sel_), CART DT model with all input variables (CART DT_all_), and CART DT model with selected input variables (CART DT_sel_) were then employed to predict outcomes. The criteria for evaluating the performance of the models were sensitivity, specificity, accuracy, F-measure, and the area under the ROC curve. Finally, the model was validated externally in poisoned patients admitted to the ICU [23]. The R program, version 4.1, was used to create AI models.

It should be noted that for the architecture of the ANN, a feedforward network with a backpropagation learning method with two fully connected hidden connected layers was used. In the end, the number of nodes in the selected ANN in the first and second hidden layers was 20 and 15, respectively. Sigmoid activity function was used in the hidden and output layers and the learning rate was set at 0.3. Also, for CART DT, Gini Index was used as the criterion for tree decomposition, and the maximum depth of the tree was limited to 5 levels.

### Statistical analysis

To carry out the statistical analysis, the data of the patients admitted to the ICU were divided into two groups with the help of the variable *“hospital mortality”*. Then, the characteristics of each group were described in the form of a frequency table. Kolmogorov–Smirnov test was used to test the normality of the data. In order to compare the studied variables in the two groups, the independent *t*-test was used in case of normality and the Mann–Whitney test was used in case of non-normality. For qualitative variables, Chi-square or Fisher test was used. SPSS Statistics 21 was used for statistical analysis, and the significance level was set at 5%.

## Results

### Overall description of data

The data were collected from 840 participating patients admitted to the ICU of Razi Medical Center in Ghaemshahr. Table 2 details the data of the patients participating in this study. This table also compares statistically the mentioned variables in the two groups of the living and the deceased. According to the table, the number of patients who died was 333 (39.6%) and the number of patients who recovered was 507 (60.4%). The mean age of the patients admitted to the ICU was 66.49 ± 17.86 years. The mean age of the deceased was 70.97 ± 14.31 years and the mean age of the recovered was 63.56 ± 19.31 years, which shows a statistically significant difference. The number of male patients participating in the study was 431 (51.3%) and the number of female patients was 394 (46.9%), the relationship of which with mortality is not statistically significant (*P* = 0.075). The average length of stay in the ICU for the patients who died was approximately 3.5 days longer than the average length of stay of the patients who recovered, which shows a statistically significant difference. Among all participants, 537 patients (63.9%) needed a mechanical ventilation device and the rest of them, i.e. 303 patients (36.1%), did not need the device.

**Table 2:** Comparison of variables in the deceased and recovered groups

Variables	Total n = 840	Dead n = 333 (39.6%)	Alive n = 507 (60.4%)	*p*-value
*Gender (%)*				
Male	431 (51.3)	182 (42.2)	249 (57.8)	0.075
Female	394 (46.9)	142 (36)	252 (64)	
*Mechanical ventilation (%)*				
Yes	537 (63.9)	222 (61.3)	315 (58.7)	0.187
No	303 (36.1)	111 (36.6)	192 (63.4)	
Age (year)	66.49 ± 17.86	70.97 ± 14.31	63.56 ± 19.31	< 0.001
LOS in ICU (day)	8.91 ± 8.06	10.33 ± 9.49	7.99 ± 6.84	< 0.001
GCS	10.97 ± 4.06	8.63 ± 4.07	12.46 ± 3.27	< 0.001
SOFA	5.45 ± 3.35	7.35 ± 3.23	4.21 ± 2.8	< 0.001
SAPS II	33.24 ± 16.08	42.49 ± 15.14	27.16 ± 13.6	< 0.001
APACHE II	18.38 ± 7.88	23.32 ± 8.22	15.13 ± 5.66	< 0.001
APACHE IV	54.88 ± 18.72	65.04 ± 17.42	48.33 ± 16.48	< 0.001
ALB	2.85 ± 0.66	2.7 ± 0.66	3.04 ± 0.62	< 0.001
HCO3	23.62 ± 7.22	22.88 ± 8.72	24.12 ± 5.97	0.033
PH	7.13 ± 0.68	7.1 ± 0.69	7.15 ± 0.67	0.308
PCO2	38.81 ± 13.72	37.11 ± 14.99	39.95 ± 12.68	0.006
FiO2	58.12 ± 29.51	54.96 ± 26.23	60.65 ± 31.7	0.038
RR	16.84 ± 6.85	16.4 ± 9.87	17.16 ± 3.72	0.194
TEMP	36.75 ± 0.45	36.76 ± 0.5	36.75 ± 0.41	0.856
HR	81.47 ± 21.49	78.83 ± 23.97	36.75 ± 0.41	0.005
SBP	113.55 ± 29.44	112.88 ± 32.07	113.99 ± 27.6	0.605
DBP	75.26 ± 19.64	76.04 ± 20.23	74.76 ± 19.24	0.355
MAP	88.02 ± 17.52	88.31 ± 19.11	87.83 ± 16.4	0.704
BS	133.99 ± 64.88	143.47 ± 76.61	127.67 ± 55.09	0.001
BUN	34.42 ± 30.38	42.62 ± 33.18	29.46 ± 27.42	< 0.001
Cr	1.53 ± 1.4	1.74 ± 1.28	1.4 ± 1.5	0.001
Na	136.29 ± 7.21	136.03 ± 8.61	136.47 ± 6.12	0.421
K	3.88 ± 0.84	3.95 ± 0.92	3.83 ± 0.79	0.063
Hct	31.39 ± 8.59	29.01 ± 9.14	33.02 ± 7.8	< 0.001
Plt	189.3 ± 104.1	166.09 ± 110.98	203.76 ± 96.95	< 0.001
Bil	1.63 ± 1.81	2.1 ± 1.66	1.14 ± 1.84	0.013
WBC	10.38 ± 5.09	10.3 ± 5.53	10.43 ± 4.79	0.737

### Variables selected for model development

Based on the results of the univariate regression test with a conventional *p*-value threshold of 0.05, fourteen variables were identified as variables related to the hospital death outcome. A full list of related and unrelated variables with the hospital death outcome is given in Table 3, along with p-values, odds ratio, and 95% CI. The fourteen variables selected to develop the AI models are age, GCS, ALB, HCO3, PCO2, HR, BS, BIL, Cr, K, Hct, PLT, BUN, and urine output.

**Table 3:** Univariate regression analysis for variable selection

Variables	Odds ratio	95% CI	*p*-value
Age	1.02	1.01–1.03	< 0.001
GCS	0.79	0.76–0.82	< 0.001
ALB	0.44	0.34–0.58	< 0.001
HCO3	0.97	0.95–0.99	0.03
PH	0.90	0.74–1.1	0.33
Mechanical ventilation	0.82	0.61–1.09	0.18
PCO2	0.98	0.97–0.99	0.005
PAO2	0.999	0.996–1.002	0.56
FiO2	1.001	0.99–1.006	0.70
RR	0.999	0.980–1.019	0.94
TEMP	1.16	0.86–1.57	0.32
HR	0.991	0.984–0.997	0.04
SBP	0.999	0.995–1.004	0.86
DBP	0.999	0.993–1.006	0.88
MAP	0.999	0.992–1.007	0.83
BS	1.004	1.003–1.006	< 0.001
BUN	1.012	1.007–1.017	< 0.001
Cr	1.16	1.063–1.266	0.001
Na	0.996	0.978–1.015	0.69
K	1.27	1.078–1.497	0.004
Hct	0.949	0.931–0.968	< 0.001
PLT	0.996	0.994–0.997	< 0.001
BIL	1.687	1.228–2.316	0.001
WBC	1.003	0.977–1.029	0.84
Urine Output	0.999	0.996–0.999	0.002
Chronic diseases/ Comorbidities	0.999	0.991–1.002	0.58

Table 3 reveals that bilirubin, potassium, and creatinine were the variables with the highest impact on hospital death. In contrast, variables related to oxygenation and blood pressure were not significantly associated with the hospital death outcome.

### Overall performances of models and sensitivity analysis

The mean and the standard deviation of the scores obtained in SOFA, SAPS II, APACHE II, and APACHE IV models, were 5.45 ± 3.35, 33.24 ± 16.08, 7.88 ± 18.38, and 54.88 ± 18.72, respectively. Also, the mean and the standard deviation of the scores for the deceased patients in the SOFA model, SAPS II model, APACHE II model, and APACHE IV model, were 7.35 ± 3.23, 42.49 ± 15.15, 8.22 ± 23.32, and 65.04 ± 17.42, respectively. In comparison to the mean scores of the patients who recovered, these scores show a statistically significant difference in all four models (*P* < 0.001).

**Fig. 1:**
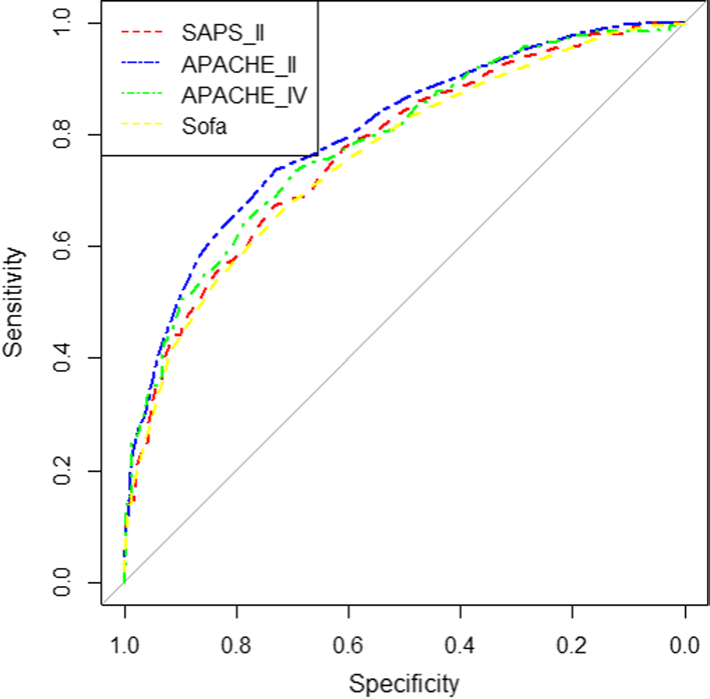
Comparison of the ROC curve in four conventional models SOFA, SAPS II, APACHE II, and APACHE IV

**Fig. 2:**
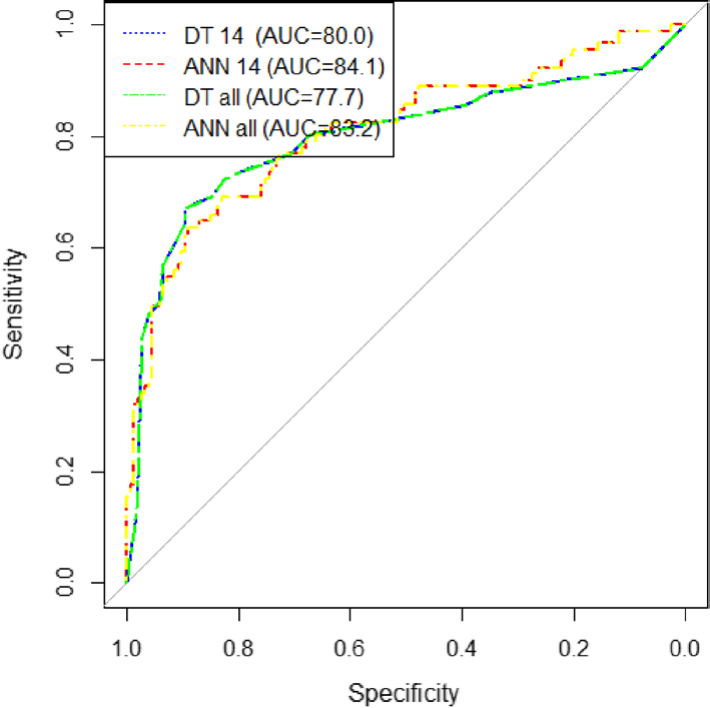
Comparison of the ROC curve in artificial intelligence models (Artificial Neural Networks and Decision Trees)

**Fig. 3:**
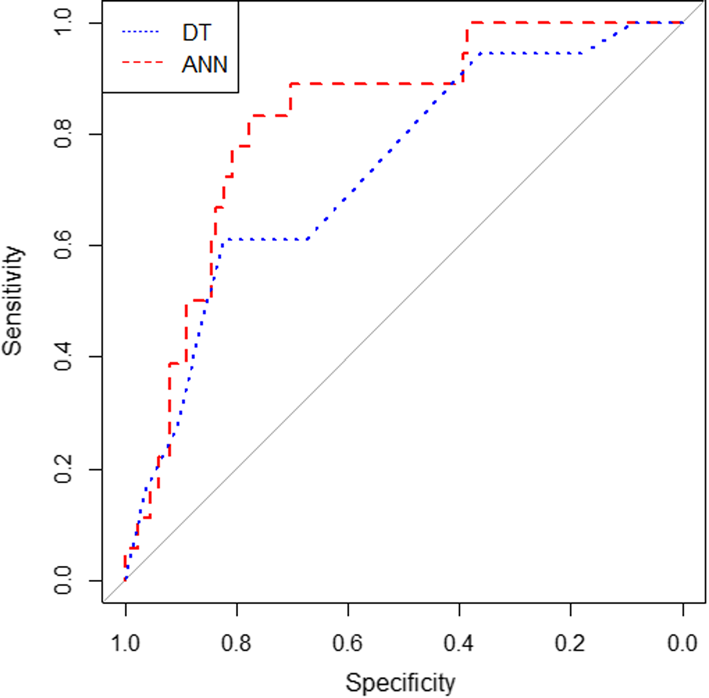
Comparison of the ROC curve in artificial intelligence models (Artificial Neural Networks and Decision Trees) for external validity

In this study, various performance evaluation indicators were used, including sensitivity, specificity, accuracy, F-measure and the area under the ROC curve. The values obtained for each of the indicators of the models are shown in Table 4. Also, the ROC curves for conventional and AI models are displayed in Figs. 1 and 2, respectively. The ROC curves obtained from the experiment of AI models on external data are depicted in Fig. 3.

**Table 4:** Sensitivity, specificity, accuracy, F-measure and area under the curve of artificial intelligence models

Model type	Name of the model	Sensitivity	Specificity	Accuracy	F-measure	Area under the ROC curve
Conventional	SOFA	66.67	71.40	69.52	63.43	76.0
	SAPS II	67.26	73.37	70.95	64.73	77.1
	APACHE II	73.9	73.0	73.3	68.71	80.3
	APACHE IV	73.6	69.4	71.1	66.86	78.5
Artificial intelligence	MLP NN_sel_	84.68	72.26	77.82	77.36	84.1
	CART DT_sel_	80.18	72.99	76.21	75.10	80.0
	MLP NN_all_	81.16	75.44	77.91	76.34	83.2
	CART DT_all_	79.81	72.34	75.6	74.03	77.7
Artificial intelligence (external validation)	MLP NN_sel_	66.67	83.70	81.69	46.15	78.9
	CART DT_sel_	83.33	51.85	55.55	30.61	72.9
	MLP NN_all_	83.3	77.8	78.43	67.63	82.3
	CART DT_all_	71.1	82.22	79.73	59.37	75.6

**Fig. 4:**
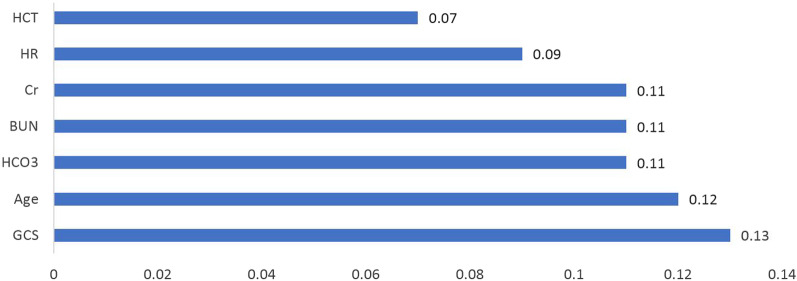
Investigation of mortality predictors in ICU with the artificial neural network model for selected variables. Variables whose importance was less than 0.05 were not shown in the chart, including PLT, BS, Urine output, PCO2, ALB, BIL and K

**Fig. 5:**
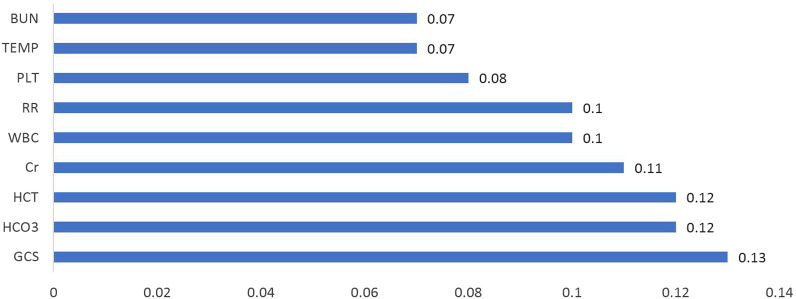
Investigation of mortality predictors in ICU with the artificial neural network model for all variables. Variables whose importance was less than 0.05 were not shown in the chart, including HR, Age, BS, BIL and Urine output

The results of the sensitivity analysis for the MLP NN_sel_ model showed that GCS, age, HCO3, BIL, Cr, HR and Hct were the most important determinants of mortality in patients admitted to the ICU (Fig. 4). However, the sensitivity analysis results for MLP NN_all_ indicate that importance of age and HR variables were 0.01 and 0.03 in predicting hospital death, respectively. Also, WBC, RR, TEMP, and PLT variables were the most important determinants of hospital death (Fig. 5). Variables whose importance was less than 0.05 were not shown in the Figs. 4 and 5.

**Fig. 6:**
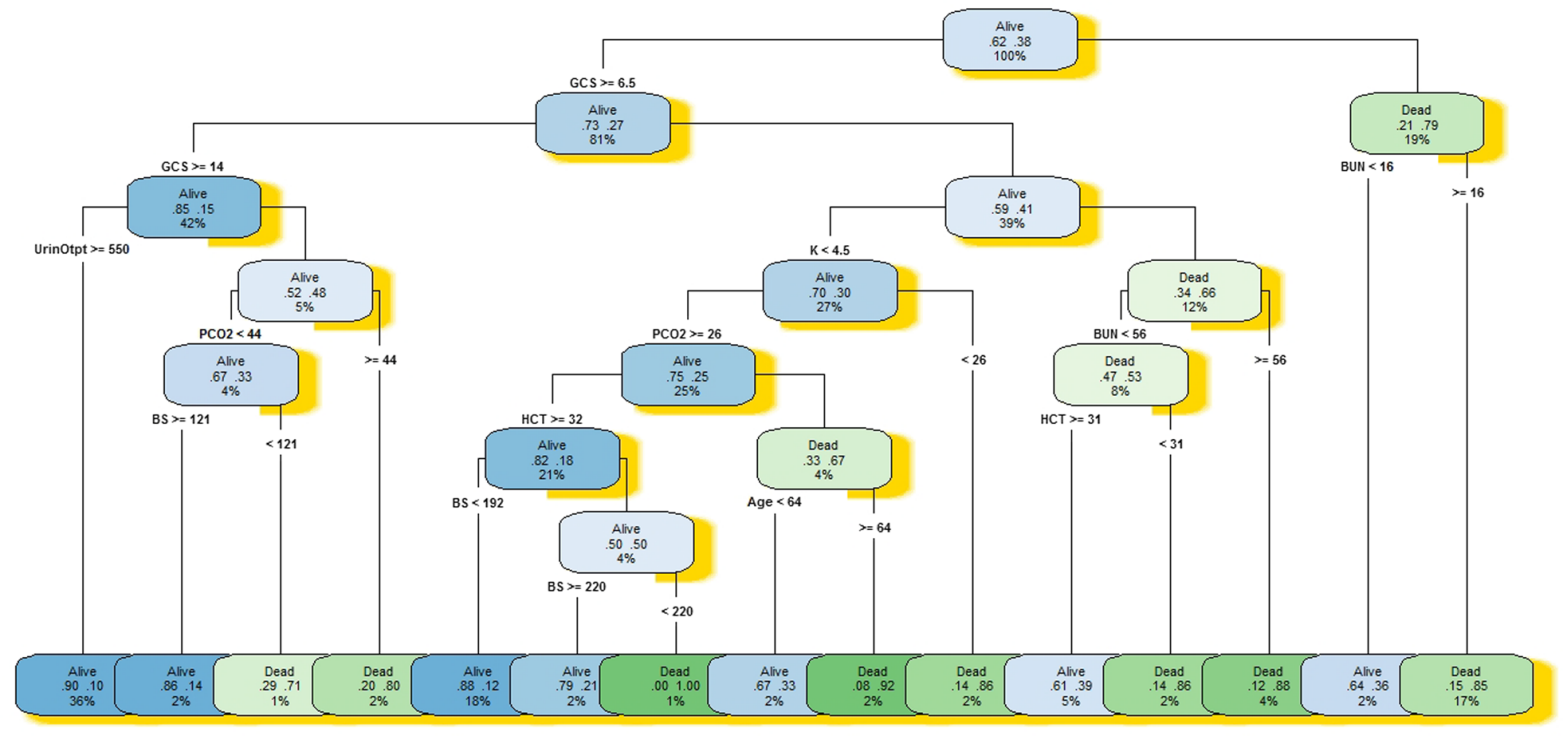
Decision tree model for predicting patient mortality in the intensive care unit for selected variables

**Fig. 7:**
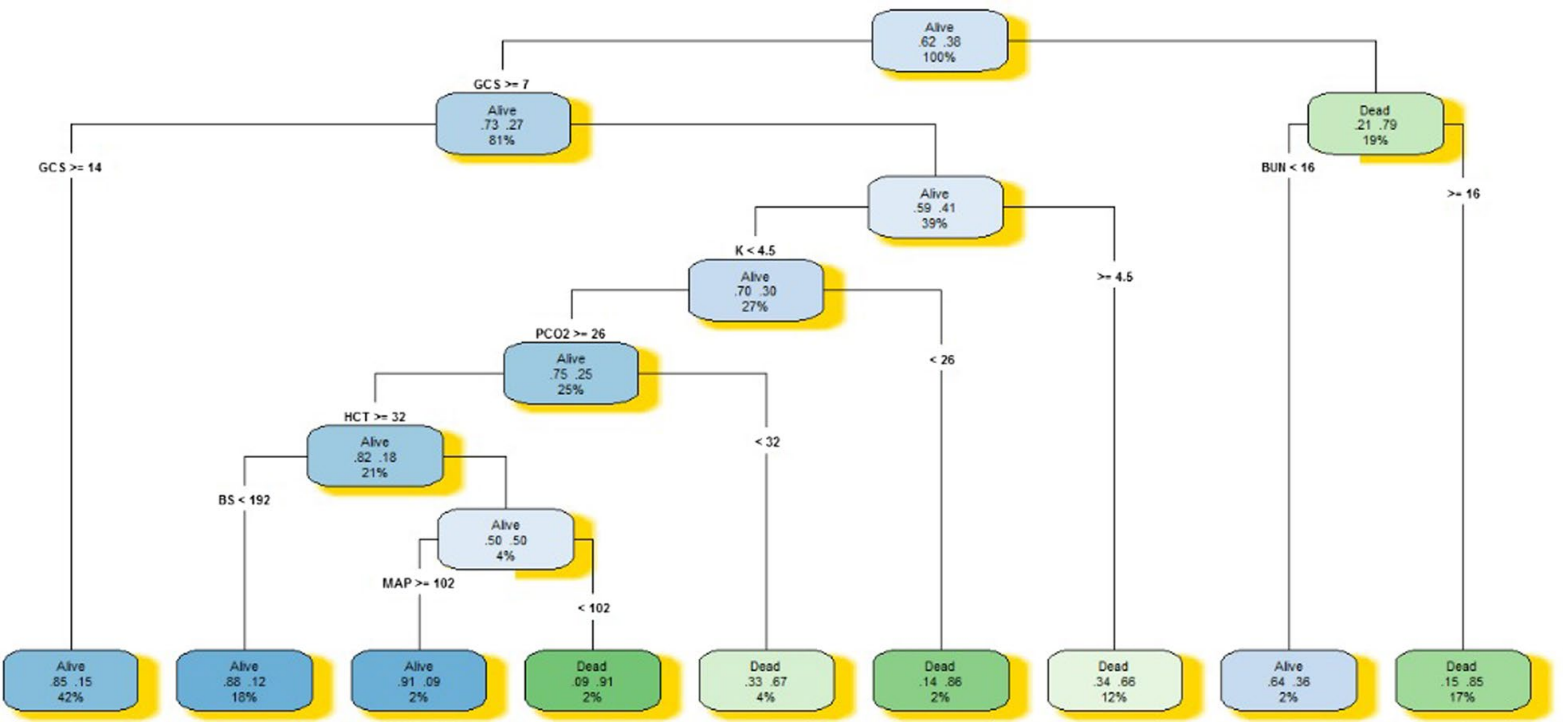
Decision tree model for predicting patient mortality in the intensive care unit for all variables

The results of the DT model showed that a patient with a level of GCS less than 6.5, a level of bilirubin higher than 16, has a 85% probability of dying. Also, a patient with a level of GCS less than 6.5, a level of bilirubin less than 16, survives with a probability of 64%. A patient with a level of GCS more than 6.5, a level of potassium more than 4.5, a level of bilirubin more than 56, dies with a probability of 88%. A patient with a level of GCS more than 6.5, a level of potassium more than 4.5, a level of bilirubin less than 56 and a level of hematocrit less than 31, has an 86% probability of dying. A patient with a level of GCS more than 6.5, a level of potassium less than 4.5, a level of PCO2 more than 26, a level of hematocrit more than 32 and a level of blood sugar less than 192, has an 88% probability of survival. And a level of GCS greater than 6.5, a level of urine output less than 550, and a level of PCO2 more than 44, dies with a 80% probability. Figures 6 and 7 present a graphical representation of the developed CART DT_all_ and CART DT_sel_.

## Discussion

According to Table 2, there is a significant relationship between predicted mortality and actual mortality, indicating that the patients who have a higher predicted risk of mortality are more likely to die. In other words, the scores for each of the models in the deceased group were higher than those of the same model in the recovered group, and the difference between the two groups was significant. Among the models, APACHE II had a better patient survival prediction. The mean score for this model was 18.38 ± 7.88; it was 23.32 ± 8.22 in the deceased group, and 15.13 ± 5.66 in the recovered group. Similarly, in a study conducted in the ICU of Namazi Hospital in Shiraz, the mean score of the APACHE II model for patients was 17.85 ± 7.4 [24]. In another study conducted in India, the mean score of APACHE II for patients was reported to be 17.8 ± 10.5, which agrees with the findings of the present study [21].

As discussed above, the second version of the APACHE scoring systems produced more accurate results than those of other conventional systems (SOFA, SAPS II, and APACHE IV). With a sensitivity of 70.62, a specificity of 81.33, and an area under the ROC curve of 0.803, this model obtained the highest values for sensitivity, specificity, and area under the curve, and provided a better performance compared to other models. The results of the present study also showed that APACHE II was followed by APACHE IV with an area under the ROC curve of 0.785, SAPS II with an area under the ROC curve of 0.771, and SOFA with an area under the ROC curve of 0.76, concerning the discrimination power in predicting the survival of patients admitted to the ICU. According to an accepted approach in data science, an area under the ROC curve above 0.75 indicates a strong and acceptable discrimination power [25]. Therefore, it can be said that all the conventional models studied in the present study have shown acceptable results in discriminating between the deceased and the recovered.

Zhu et al. demonstrated that APACHE II outperformed SOFA regarding in-hospital mortality prediction, with respective cut-off points of 17 and 3 [26]. Colussi et al. demonstrated that SAPS II with a cut-off point of 49 and an 82% area under the ROC curve was superior than APACHE II with a cut-off point of 22 and a 76% area under the ROC curve in predicting septic disease. The model with the lowest performance was SOFA, which had a cut-off point and area under the ROC curve of 65% [27].Wang et al. in a large prospective multicenter trial compared the capability of APACHE II, SAPS II, and SOFA scores in predicting 28 days mortality in AKI patients. They observed that SAPS II functioning was superior, followed by that of APACHE II. However, the difference between the three scoring systems was not significant. The SOFA score had the least predictive value [28]. In an epidemiological research and clinical observation, according to the APACHE II score and the mean SOFA value of admission days, the mortality rate for ICU was 21.5 and 7.3%, respectively. The mean daily SOFA score exhibited a stronger predictive performance (*P* < 0.001) with a cut-off point of 13 for APACHE II and 5 for the SOFA score [29]. In another study, Costa e Silva et al. reported that SOFA with a cut-off point of 4 and a 75% area under the ROC curve could more accurately predict mortality of liver cirrhosis patients than APACHE II with a cut-off point of 17 and the area under the ROC curve of 69% [30]. According to Huang et al., SAPS II with an area under the ROC curve of 71% performed better than SOFA with an area under the ROC curve of 66% in predicting the mortality of patients with acute respiratory distress syndrome [31]. In another study, the prognostic significance of APACHE II and IV scores versus SOFA scores in admission to ICU mortality was investigated in a cohort study of ICU patients with severe SARS-CoV-2 pneumonia. APACHE IV values were more accurate at predicting ICU mortality than SOFA scores. In addition, the APACHE IV intensity scoring system had the greatest calibration values in comparison to APACHE II and SOFA [32]. In Schoe et al.’s study, only the SAPS II model exhibited adequate calibration for measuring the result of ICU mortality in patients who underwent cardiac surgery in the ICU. APACHE IV and APACHE II models with 91% and 89% showed lower performance regarding the area under the ROC curve. The SOFA model performed the worst, with an area under the ROC of 86% [33].

In the present study, as in some other studies [20, 23, 34], SOFA showed the least discrimination power in distinguishing between the deceased and the recovered. This could be because this model has been primarily designed for a purpose other than predicting patient survival. In fact, this model has been created to determine the severity of changes in organ failure in patients with sepsis admitted to the ICU. Therefore, this model seems to be more suitable for predicting morbidity [35]. However, the present study results showed that it could also be used to predict the survival of patients admitted to the ICU. Thus, since the performance of SOFA does not show a clinically significant difference from that of other conventional models, and since the number of variables used in this model is much less than the number used in other conventional models, the use of this model is recommended for predicting the survival of patients admitted to the ICU.

The results of the AI models showed that the MLP NN_sel_, with a sensitivity of 84.68, a specificity of 72.26, and an area under the ROC curve of 84.1, outperformed other AI models. In all AI models, the sensitivity value was greater than the specificity value. Therefore, they are more capable of predicting patient survival. However, in SOFA and SAPS II, the specificity values were higher than the sensitivity values, indicating that these two models can better distinguish between the deceased and the recovered cases.

The results of the ANN and DT models revealed that these new models have the power to compete with conventional models in predicting ICU patient survival and yield more acceptable results. The present study results indicated that although MLP NN_sel_ had the best performance among all models (including conventional models and DT models), MLP NN_all_ outperformed all models in external validation. Also, MLP NN_all_ did not show a clinically significant difference with MLP NN_sel_ in terms of the area under the ROC curve. In an Indian psychiatric ICU research study comparing APACHE II and an ANN, the area under the ROC curve of the two models was reported to be 0.77 and 0.87, respectively [21], which are superior to our results. This discrepancy can be attributed to the difference between the patients in these two studies since the present study was performed in a general ICU. In contrast, the study in India was performed with the patients in a psychiatric ICU. In another study carried out on the patients with pneumonia admitted to the ICU, the DT model was compared with conventional models, SOAR and CURB-65, in terms of performance [19]. The results of this study, unlike our findings, revealed that the DT model (sensitivity of 73.4, a specificity of 49.00, and an area under the ROC curve of 0.661) performed better than the conventional models CURB-65 (sensitivity of 74.5, a specificity of 42.3, and an area under the ROC curve of 0.608) and SOAR (sensitivity of 0.589, a specificity of 80.7, and an area under the ROC curve of 0.589). The difference between the results of this study and our study can be attributed to the fact that the present study was performed in a ‘general’ or regular ICU, while the above study was carried out in a ‘specialty’ ICU.

Frize et al. investigated 1,491 ICU patients in Canada. They utilized two-thirds of these patients’ data for training the ANN, while the remaining one-third was used for validation. In this study, the ANN model and APACHE II showed equal accuracy in predicting outcomes. Nonetheless, the ANN was able to predict the outcome using only six of the characteristics employed by APACHE II [36]. In another study from the United Kingdom, the Trauma and Injury Severity Score (TRISS) model and an ANN were used to compare the anticipated outcomes of trauma patients. In this study, TRISS demonstrated superior discrimination, whereas the ANN exhibited superior goodness-of-fitness/calibration. The researchers discovered that the TRISS model, which posits a linear connection between predictor variables and outcomes, outperformed the ANN regarding discrimination. However, the ANN dealt with nonlinear variables more effectively and had a more accurate calibration than the TRISS model. Similar to our findings, these researchers discovered that the ANN could predict outcomes using fewer factors [37].

There are probable causes for the ANN models’ apparent advantage over the conventional models in our patients. Although specialists score the factors of conventional models, the final mortality prediction equation in conventional models is constructed using logistic regression, implying a nonlinear connection between predictor variables and outcomes. ANNs are efficient in developing nonlinear models and may thus provide a theoretical benefit. Significant differences exist between the patient populations of Iranian, European, and American ICUs, which may have impacted the accuracy of conventional models. Iranian ICU patients differ from American and European ICU patients concerning other outcome-influencing variables, such as lead-time bias.

Another notable finding in our study was that several of the variables employed by the conventional models were redundant. Indeed, they did not improve prediction accuracy and could be removed from the model-building process.

Based on Table 3, the variables PH, mechanical ventilation, PAO2, Fio2, RR, Temp, SBP, DBP, MAP, Na, WBC, and chronic disease did not have informational value in predicting mortality. Similarly, Wong and Young managed to delete several factors from the APACHE II model without sacrificing accuracy [37]. Also, Frize et al. could predict outcomes with ANNs using only six of the APACHE II system’s parameters, suggesting that although ANN models might be just as effective as or even better than APACHE II at predicting outcomes, their greatest strength may lie with their capacity to do so with fewer variables [36]. Clermont et al. discovered that outcome prediction was accurate even after excluding factors such as initial diagnosis and location before ICU admission [38].

Although the variables WBC, RR, and TEMP were not statistically related to nosocomial mortality, as shown in Table 3, the sensitivity analysis findings for MLP NN_all_ indicate that these three factors are significant in predicting nosocomial death. Possibly this can be explained by the interplay of factors with one another. This indicates that when the variables are positioned adjacent to one another, the interaction between them creates a significant relationship that cannot be detected by the statistical univariate test.

The results of sensitivity analysis of the MLP NN_sel_ model showed that GCS, age, HCO3, BUN, Cr, Hr and Hct are the most important determinants of mortality in ICU patients, respectively. In line with the results of the present study, Asgari reported that glucose, relative thromboplastin time, white blood cells, systolic blood pressure, creatinine, albumin, and bilirubin are the most effective variables in predicting mortality in the ICU [39]. Barfod et al. introduced some vital signs, including peripheral oxygen saturation, RR, SBP, and GCS, as the predicting factors of mortality in the ICU. Based on the results of this study, not only the type but also the number of abnormal vital signs were predictive of adverse outcomes. The chief complaints associated with high in-hospital mortality were shortness of breath and altered level of consciousness [40]. The use of vital signs to predict mortality has been reported to be effective in other studies as well [41, 42]. GCS is also a physiological scoring system that is considered an important criterion for neurological evaluation. In previous studies, low GCS has been reported to be associated with poor prognosis [43, 44]. However, the measurement of GCS can be complicated in some cases. It is difficult to assess this scale in cases where the patient is intubated, has received sedation, is poisoned, or has jaw or facial injuries [43]. Since most of the mortality predicting variables in this study include vital signs and routine medical tests which are routinely recorded, the risk of patient mortality can be measured continuously and automatically.

There are a number of limitations to this study. The first limitation is that the study was conducted in one medical center; therefore, the findings cannot be generalized to other populations. Nonetheless, the results might be more generalizable if the study was conducted at more centers. The second restriction is the retrospective nature of the present investigation. Therefore, it is recommended that future research in this domain be conducted prospectively to avoid the limitations of retrospective studies. Although the ANN fared best in forecasting hospital mortality, it is difficult to comprehend how factors contribute to death prediction since the neural network functions like a black box. Consequently, it might be difficult to convince clinicians to utilize an ANN to forecast hospital mortality.

## Conclusion

The ANN model has more predictive power than other conventional models in predicting patient survival in the ICU and can be used as an alternative to conventional models. To better evaluate the performance of the models used in this study, it is suggested that future research be conducted prospectively to remove limitations such as the existence of missing data.

## Data Availability

The datasets analyzed during the current study are not publicly available due to privacy but are available from the corresponding author on reasonable request.

## Abbreviations

APACHE: Acute physiology and chronic health enquiry
SAPS: Simplified acute physiology score
SOFA: Sequential organ failure assessment
GCS: Glasgow coma score
SBP: Systolic blood pressure
DBP: Diastolic blood pressure
MAP: Mean arterial pressure
HR: Heart rate
RR: Respiratory rate
PH: Arterial pH
Cr: Serum creatinine
Na: Serum sodium
K: Serum potassium
BUN: Blood urea nitrogen
HCO3: Bicarbonate
BIL: Bilirubin level
BSL: Blood sugar level
WBC: White blood count
PLT: Platelets
Hct: Hematocrit
AI: Artificial intelligence
ANN: Artificial neural network
DT: Decision tree
MLP: Multi layer perceptron
ICU: Intensive care unit
